# The Abbreviated Overall Anxiety Severity and Impairment Scale (OASIS) and the Abbreviated Overall Depression Severity and Impairment Scale (ODSIS): Psychometric Properties and Evaluation of the Czech Versions

**DOI:** 10.3390/ijerph181910337

**Published:** 2021-09-30

**Authors:** Jan Sandora, Lukas Novak, Robert Brnka, Jitse P. van Dijk, Peter Tavel, Klara Malinakova

**Affiliations:** 1 Olomouc University Social Health Institute, Palacky University Olomouc, 771 11 Olomouc, Czech Republic; lukas.novak@oushi.upol.cz (L.N.); robert.brnka@gmail.com (R.B.); j.p.van.dijk@umcg.nl (J.P.v.D.); peter.tavel@oushi.upol.cz (P.T.); klara.malinakova@oushi.upol.cz (K.M.); 2 First Department of Internal Medicine, Faculty of Medicine, Comenius University Bratislava, 813 72 Bratislava, Slovakia; 3 Department of Community and Occupational Medicine, University Medical Center Groningen, University of Groningen, 9713 AV Groningen, The Netherlands; 4 Graduate School Kosice Institute for Society and Health, P.J. Safarik University in Kosice, 040 11 Kosice, Slovakia

**Keywords:** OASIS, ODSIS, anxiety, depression, COVID-19, Czech versions, psychometric properties

## Abstract

Short and effective tools for measuring depression, anxiety and their resulting impairments are lacking in the Czech language. The abbreviated versions of the Overall Anxiety Severity and Impairment Scale (OASIS) and the Overall Depression Severity and Impairment Scale (ODSIS) show very good psychometric properties in English and other languages, and can be used in different settings for research or clinical purposes. The aim of this study was the psychometric evaluation and validation of the Czech versions of the abbreviated forms of both tools in the general population. A nationally representative sample of 2912 participants (age = 48.88, *SD* = 15.56; 55% female) was used. The non-parametric testing of the differences between sociodemographic groups revealed a higher level of anxiety and depression in students, females and religious respondents. Confirmatory Factor Analysis suggested a good fit for the unidimensional model of the OASIS: *x*^2^(4) = 38.28; *p* < 0.001; *TLI* = 0.999; *CFI* = 0.997; *RMSEA* = 0.078; *SRMR* = 0.027 and the ODSIS: *x*^2^(4) = 36.54; *p* < 0.001; *TLI* = 0.999; *CFI* = 0.999; *RMSEA* = 0.076; *SRMR* = 0.021 with the data. Both scales had an excellent internal consistency (OASIS: Cronbach’s alpha = 0.95, McDonald’s omega = 0.95 and ODSIS: Cronbach’s alpha = 0.95, McDonald’s omega = 0.95). A clinical cut-off of 15 was identified for the OASIS and a cut-off of 12 for the ODSIS. The study showed good validity for both scales. The Czech versions of the abbreviated OASIS and ODSIS were short and valid instruments for measuring anxiety and depression.

## 1. Introduction

Mood disorders and anxiety play an increasingly significant role in the mental health of the general population. Mass traumatic events, such as natural disasters or pandemics, both of which we are witnessing on an unprecedented level, threaten and influence many individuals’ mental health and satisfaction of basic human needs and goals [1,2,3]. According to the World Health Organization [4], in 2015 the proportion of the worldwide population affected by depression amounted to 4.4%. In the same year, the proportion of the population with anxiety was assessed to be 3.8% [4]. In the Czech Republic, the level of depression was estimated to be even higher, at 5.8%, and the level of anxiety was the same as that of the worldwide population. A systematic review by Xiong et al. [5] found relatively high rates of anxious and depressive symptoms in the general population due to psychological distress caused by the COVID-19 pandemic. Consequently, due to the ongoing pandemic situation, we can assume that all these levels are higher than usual in the Czech population as well.

Assessing the level of depression and anxiety in various settings, both clinical and non-clinical, can prove valuable and necessary in order to provide interventions or to understand, explain or predict human behavior. Especially in research settings, where a larger number of tests are being distributed, it is important to have a short tool able to assess the targeted symptoms. A comprehensive overview of the available self-reported and clinician-administered measures for assessing anxiety is provided by Antony et al. [6]. These measures focus on anxiety and its related constructs (e.g., Beck Anxiety Inventory (BAI) [7] or Positive and Negative Affect Schedules (PANAS) [8]), as well as on specific diagnostic categories (e.g., the Panic and Agoraphobia Scale (PAS) [9]). Additionally, a variety of measures focus on assessing depression, such as the Beck Depression Inventory-II (BDI-II) [10] or the Patient Health Questionnaire-9 (PHQ-9) [11]. Many of these measures include only a few items and take 5–10 min to complete. However, in clinical and in research settings, it is often especially important to use a broad, but not time-consuming, assessment tool suitable for a quick screening aimed not only at symptoms, but also at the functional and behavioral impairment resulting from anxiety and mood disorders.

In order to meet these criteria, the Overall Anxiety Severity and Impairment Scale (OASIS) was developed and validated in English by Norman et al. [12]. The OASIS is a short, five-item self-report questionnaire designed to identify individuals with anxiety-related problems and to assess the frequency and intensity of the associated symptoms, as well as the functional impairment of the individual during the period of the past week. The original measure uses long descriptions stating the frequency of the symptoms as responses, followed by an elucidation of the possible accompanying symptoms and their intensity in more detail to elaborate on how to understand the answer. The answers also include examples of possible beavioral and emotional consequences. Using this original measure may be more time-consuming or demanding. Thus, the authors themselves introduced an abbreviated version of the OASIS [13] which uses only short, one-to-three-word responses and five answers per question to identify the frequency of the symptom in question. Norman et al. [12] reported that the OASIS showed excellent convergent validity with the Brief Symptom Inventory 18 (BSI-18), the Spielberger Trait Anxiety Questionnaire and the Back Depression Inventory (BDI), as well as strong test–retest reliability. The OASIS was also validated in different cultural and language environments. There is a Spanish version [14,15], a Dutch version [16], a Japanese version [17] and a Persian version [18]. All studies confirm the good psychometric properties of the OASIS and support its use in different settings.

In order to provide a tool for assessing depression in a similar manner, Bentley et al. [19] developed the Overall Depression Severity and Impairment Scale (ODSIS) by directly modifying the OASIS. The ODSIS has the same five-item structure as the OASIS and, analogously, it captures the severity and functional impairment resulting from depressive symptomatology over the past week. Bentley et al. [19] reported a very good convergent validity of the ODSIS with the pre-existing measures of depression, such as the Beck Depression Inventory-II (BDI-II), the Center for Epidemiologic Studies Depression Scale (CES-D), the Depression Anxiety Stress Scale (DASS-D) and the Patient Health Questionnaire-9 (PHQ-9). The evaluation of the test–retest reliability was not completed in the original study. The ODSIS had a validated Japanese version [20] and a Spanish version [15,21]. Similar to Norman et al. [13], the study of Ito et al. [20] used and validated an abbreviated version of the ODSIS. The ODSIS shows good psychometric properties across the above-mentioned studies and is suitable for use in different settings. 

As the OASIS and the ODSIS are short, accurate and time-effective assessment and screening tools for anxiety and mood disorders which proved to be reliable and valid in different countries, they can also contribute substantially to the scientific and clinical work in the Czech language environment. The goal of the present study is to adapt the abbreviated versions of the OASIS and the ODSIS as two brief tools for assessing anxiety and depression in the Czech scientific environment, to carry out a psychometric evaluation of the Czech version of both tools, and to provide further evidence for the psychometric soundness and transcultural validity of both scales.

## 2. Materials and Methods

### 2.1. Participants and Procedure

In the present study, four different samples were used. The first and the second sample were collected in the general Czech population, aged 18 to 97 years, during the first wave of the COVID-19 pandemic and the first lockdown from 1 April 2020 to 1 May 2020 (Sample 1, *n* = 1393) and from 27 May 2020 to 23 June 2020 (Sample 2, *n* = 1015) by means of an online survey. The survey was designed at the researchers’ institution. A professional agency (The Czech National Panel, Prague, Czech Republic) distributed the survey and collected the data in order to achieve a balanced sample regarding age and gender, close to national representative characteristics.

As the survey was administered only once and the subjects were not retested, it was necessary to collect additional data in order to establish test–retest reliability. For this purpose, a small sample (Sample 3, *n* = 10) was used to assess the test–retest reliability. The data were collected between 11 January 2021 and 19 January 2021. To determine the cut-off value, data from a fourth sample (Sample 4, *n* = 494) were used. The data were collected between 10 October 2020 and 5 February 2021 using the snowball sampling method.

Next, an extremely short response time and a unified pattern of responses were used as exclusion criteria and applied to the collected data. Thus, after the exclusion of participants responding inconsistently (*n* = 27) and low quality respondents (*n* = 78), the final sample (samples 1 + 2 + 3 + 4) consisted of 2912 participants (age = 48.88, *SD* = 15.56; 55.0% females). Before completing the surveys, the participants received written information regarding the purpose of the study and the anonymization of the data. Participation in the study was voluntary; the participants were allowed to leave the survey at any time without having to complete it or to state a reason for leaving. Participants were asked to explicitly express their informed consent prior to the study. The study design was approved by the Ethics Committee of the Faculty of Theology of Palacký University in Olomouc, Czech Republic (No. 2020/06).

### 2.2. Measures

The Czech versions of the abbreviated OASIS and ODSIS were obtained using the forward- and back-translation procedure. First, both scales were translated into Czech by two independent native-speaker translators and compared. The differences were discussed in a working group consisting of the researchers and the translators in order to acquire a unified version of the tools. The integrated versions were then professionally translated back into English by a native English translator fluent in the Czech language. The translations were compared with the original scales. Consequently, the differences were discussed and the final versions were agreed upon.

#### 2.2.1. The Overall Anxiety Severity and Impairment Scale (OASIS)

Anxiety was measured using the abbreviated version of the OASIS, a short 5-item self-report tool [13]. Its items measured the frequency and severity of anxiety symptoms, avoidance, home/school/work and social interference caused by anxiety. Each item required respondents to choose one of five responses which best illustratedd their experience of the described symptoms over the past week. The reponses ranged from 0 (Never) to 4 (Constantly/Extreme/All the Time) and could be added to create an overall score, which ranges from 0 to 20. Higher scores suggested a higher impairment. The cut-off score for the original language version of the OASIS was identified in different settings to be ≥8 [13,22,23], correctly identifying individuals with an anxiety disorder. A study by Bragdon et al. [24] differentiated the cut-off score on a sample of psychiatric outpatients ranging from ≥4 (at least moderate severity) to ≥12 (severe or greater illness). The cut-off scores of the versions in other languages were inconsistent and varied from 5 [16] to 10 [15].

#### 2.2.2. The Overall Depression Severity and Impairment Scale (ODSIS)

Depression was assessed using the ODSIS, a short 5-item self-report measure [19], an instrument used to assess the severity of depressive symptoms and the resulting impairment, similar to the OASIS. The responses were coded and interpreted in the same way as with the OASIS. The optimal cut-off score for the original version was identified to be ≥8 [19]. The Japanese version [20] had an optimal cut-off score ≥11. The studies validating the Spanish version identified a cut-off score of 5 with the online form [21] and a cut-off score of 10 with the regular version [15].

#### 2.2.3. Big Five Inventory, Neuroticism (BFI-N)

Neuroticism was measured using the Big Five (BFI-N), a widely accepted and validated five-factor model of personality consisting of the factors: Extraversion, Neuroticism, Conscientiousness, Agreeableness, and Openness to Experience [25]. The Neuroticism factor (N) determined the level of emotional instability and adaptation [26]. The BFI-N was a self-report tool consisting of 8 items. The respondents chose one of five answers ranging from “Fully disagree” (1) to “Fully agree” (5). The internal consistency of the Czech BFI-N was sufficient: Cronbach’s *α* = 0.87, 95%CI (0.86–0.88) and McDonald’s *ωt* = 0.87, 95%CI (0.85–0.88).

#### 2.2.4. Rosenberg Self-Esteem Scale (RSES)

Self-Esteem was measured using the Rosenberg Self-Esteem Scale [27]. This was a 10-item tool used for measuring self-esteem, with a score ranging from 0 to 30, with a higher score indicating a higher self-esteem. The items included positive and negative statements about oneself. The internal consistency of the Czech RSES was: Cronbach’s *α* = 0.86, 95%CI (0.84–0.87) and McDonald’s *ωt* = 0.86, 95%CI (0.85–0.87).

#### 2.2.5. The Positive and Negative Affect Schedule (PANAS)

One’s positive and negative affects were measured by the Positive and Negative Affect Schedule [8]. This was a 20-item self-report tool assessing positive (PANAS-P) and negative (PANAS-N) affects. Respondents rated one-word items describing positive or negative feelings and emotions on a scale from 1 to 5. The score of each subscale ranges from 10 to 50. Higher scores indicated higher levels of the respective affects. The internal consistency of the Czech version of the PANAS-P was: Cronbach’s *α* = 0.92, 95%CI (0.91–0.92) and McDonald’s *ωt* = 0.91, 95%CI (0.91–0.92). The internal consistency of the PANAS-N was: Cronbach’s *α* = 0.94, 95%CI (0.93–0.94) and McDonald’s *ωt* = 0.94, 95%CI (0.94–0.94).

#### 2.2.6. General Anxiety Disorder-7 (GAD-7)

General Anxiety was measured with the General Anxiety Disorder-7 [28]. This was a self-administered screening tool consisting of 7 items designed to identify the presence of a clinically significant general anxiety disorder. The items were decoded from 0 to 3 (0 = not at all, 1 = several days, 2 = more than half the days, 3 = nearly every day), resulting in a score ranging from 0 to 21. The cut-off points of 5, 10 and 15 indicated the presence of mild, moderate or severe levels of anxiety. The internal consistency of the Czech version of the GAD-7 reached: Cronbach’s *α* = 0.86, 95%CI (0.64–0.96) and McDonald’s *ωt* = 0.87, 95%CI (0.64–0.96).

#### 2.2.7. Patient Health Questionnaire-9 (PHQ-9)

Depression was also measured with the Patient Health Questionnaire-9 (PHQ-9), which was the depression module of the PHQ [11]. It was a 9-item self-report tool measuring depression. Items were coded from 0 to 3 (0 = not at all, 1 = several days, 2 = more than half the days, 3 = nearly every day), scoring each of the nine Diagnostic and Statistical Manual of Mental Disorders (DSM)-IV criteria for depression. The total score ranges from 0 to 27. The level of depression severity could be assessed as minimal (score 1 to 4), mild (score 5 to 9), moderate (score 10 to 14), moderately severe (score 15 to 19) or severe (score 20 to 27). Major depression could be diagnosed if five or more items were coded as 2 or higher and if one of the presented symptoms was a depressed mood or anhedonia. The internal consistency of the Czech version of the PHQ-9 reached: Cronbach’s *α* = 0.86, 95%CI (0.63–0.95) and McDonald’s *ωt* = 0.87, 95%CI (0.63–0.95).

### 2.3. Statistical Analyses

Mardia’s test of skewness and kurtosis were used to examine data distribution. Inspection of residual plots suggested a slight heteroscedasticity. Outlying scores on the OASIS and the ODSIS were identified by the Median Absolute Deviation (MAD). As our data were not normally distributed, Spearman rank correlation and non-parametric group comparison tests were used to explore relationships between variables of interests. In the two analysis, due to low statistical power, Pearson correlations were used. Effect size for non-parametric group comparison (Dunn and Games-Howell tests) was estimated by Vargha and Delaney *Ȃ* [29], where values of *Ȃ* between 0.56–0.64 indicated a small effect size; 0.64–0.71 a medium effect size and above 0.71 a large effect size. Known-group validity was examined by Analysis of Covariance (ANCOVA). Convergent validity was explored by correlations; see the preregistration form [30] for more details. The factorability of the data was explored by the Bartlett’s test and by Kaiser–Meyer–Olkin (KMO).

A significant Bartlett’s test and KMO values above 0.7 indicated that the variables were adequately correlated [31]. Calculation of the required sample size for Confirmatory Factor Analysis (CFA) revealed that at least 1021 subjects were needed; see [30] for more details. Model fit was evaluated by the following parameters: (a) Standardized Root Mean Square Residual (*SRMR*), in which values <0.08 indicated an acceptable fit and <0.05 a good fit [32]; (b) Root Mean Square Error of Approximation (*RMSEA*), in which values <0.08 were acceptable [33,34,35]; and (c) Comparative Fit Index (*CFI*) and the Tucker–Lewis Index (*TLI*). In both values, >0.95 suggested an acceptable fit [36] and values >0.97 a good fit [37]. The Chi-Square test was also calculated to compare the sample implied and observed covariance of the sample. As a fitting algorithm, the Diagonally Weighted Least Squares estimator (DWLS) was used on a matrix of polychoric correlations. A one-factor model was estimated in the OASIS and the ODSIS across all CFAs for these two reasons: first, both scales were developed as unidimensional; second, any solution other than a unidimensional solution would result in less than 3 items per factor, which was considered as insufficient for the appropriate representation of a construct [38]. For the latter reason, we did not perform Exploratory Factor Analysis (EFA). A Chi-Square difference test with Satorra–Bentler correction was used to compare the nested models. In order to explore the robustness of the factor analytic findings, we performed CFA with the first two samples. In the first sample (*n* = 1017), the one-factor model fit was evaluated. On the second sample (*n* = 1418), we aimed to replicate our CFA results and also explore configural, metric, scalar and error invariance between the two genders. A decrease in *CFI* >0.01 was used as a cut-off to compare the fit of the models during measurement invariance testing. The CFA parameters were estimated in the lavaan package [39] in the R programming software [40]. On the second sample, we performed the rest of our analysis except test–retest reliability, which was completed on a separate sample. Receiver Operating Characteristic (ROC) and Youden index were used to estimate sensitivity, specificity and optimal cut-off. Test–retest reliability was explored by intraclass correlations. The time between the first and the second administration was one week. Internal consistency was explored using Cronbach’s alpha and McDonald’s omega. For reliability estimates and other analyses, the following R packages were used: mice [41], papaja [42], psych [43], usf [44], and ICC.Sample.Size [45].

## 3. Results

### 3.1. Sociodemographic Results

The first sample (mean age = 49.62, *SD* = 16.67; 50.0% females), as well as the second sample (mean age = 48.29, *SD* = 16.42; 50.0% females), were balanced in terms of gender. See Table 1 for more details about the two samples. The retest third sample (mean age = 32.2, *SD* = 10.89; 70.0% females) was composed primarily of females who had completed university education and were employed and married. For the definition of cut-off points, sensitivity and specificity, we used a fourth sample (Age: *M* = 32.38, *SD* = 11.01; 55.0% females).

The Dunn and Games-Howell tests indicated that a higher anxiety was observed in students compared to employed/entrepreneurs, and compared to pensioners in the second sample. Compared to the non-religious, higher anxiety was also reported in religious participants, both members and non-members of a church. Similarly, a significantly lower degree of anxiety was found in atheists when compared to religious participants who were members of a church, compared to those who were not members of a church.

Similar trends were found for depression: a significantly elevated ODSIS score was reported in students compared to employed/entrepreneurs, or compared to pensioners. Higher depression was also reported in the religious participants who were not members of a church, as compared to the non-religious. No other significant differences were observed between sociodemographics of the groups.

### 3.2. Confirmatory Factor Analysis Results

**Figure 1:**
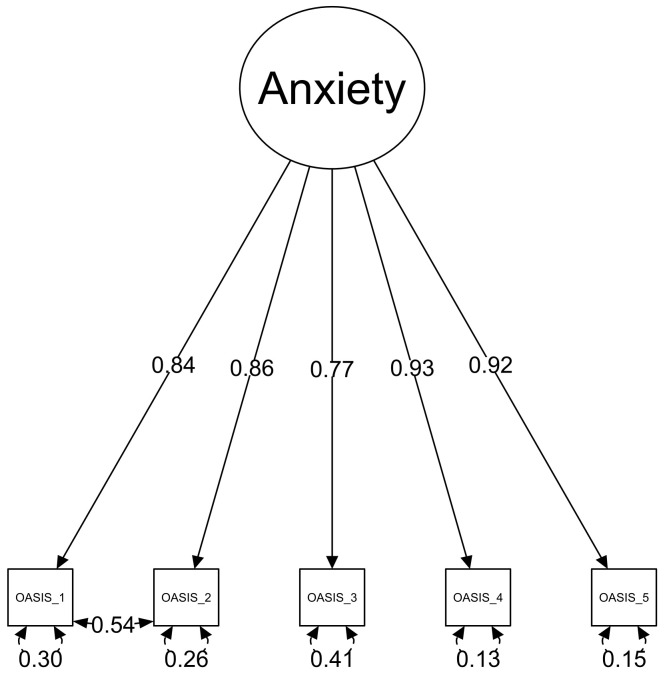
Factor loadings and residuals of the individual items of the OASIS, with correlated error terms between item 1 and 2 (Sample 2, *n* = 1015).

A significant Bartlett’s test and the lowest KMO-values of 0.83 in both datasets supported the factorability of the data. The CFA of the OASIS performed on the first sample (*n* = 1015) indicated that a one-dimensional solution did not sufficiently fit the data: *x*^2^(5) = 81.9, *p* < 0.001, *CFI* = 0.997, *TLI* = 0.995, *SRMR* = 0.036, *RMSEA* = 0.126, 90%CI (0.103–0.151). Based on the approach used in previous validation studies of the OASIS [15,17,22], we correlated error terms between items 1 and 2 to increase the model fit (see Figure 1.). As a result of this adaptation, the model fit significantly improved with *x*^2^(4) = 19.01, *p* < 0.001, *CFI* = 0.999, *TLI* = 0.999, *SRMR* = 0.021, *RMSEA* = 0.062, 90%CI (0.036–0.092). The superiority of the latter model was supported by a significant Chi-Square difference test: *x*^2^(1) = 78.95; *p* < 0.001. The highest correlation between the residuals of the manifest variables was *r* = 0.06.

**Figure 2:**
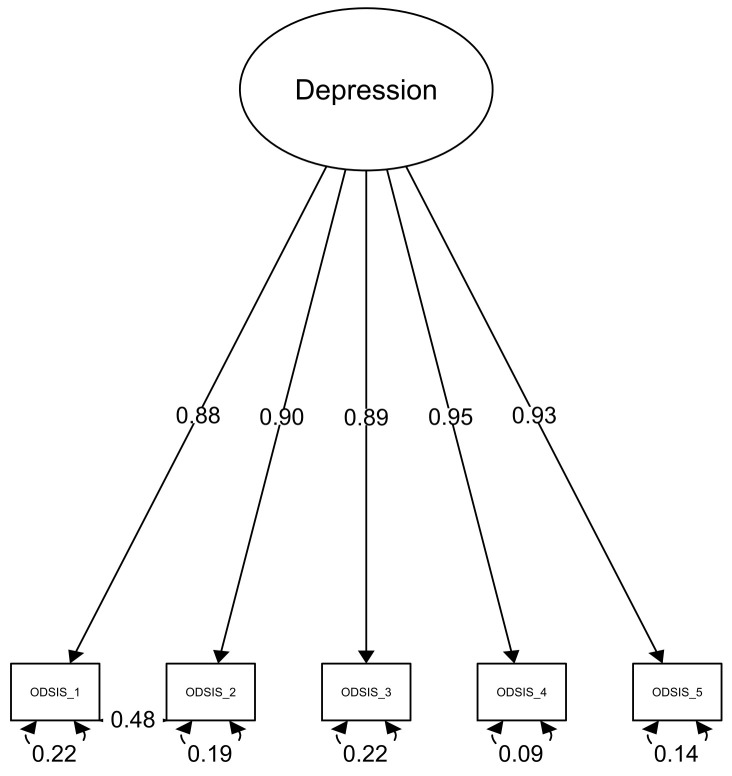
Factor loadings and residuals of the individual items of the ODSIS, with correlated error terms between items 1 and 2 (Sample 2, *n* = 1015).

Regarding the ODSIS, the originally proposed model did not fit the data well: *x*^2^(5) = 64.18, *p* < 0.001, *CFI* = 0.999, *TLI* = 0.997, *SRMR* = 0.029, *RMSEA* = 0.111, 90%CI (0.087–0.135). Thus, based on the approach used in previous validation studies of the ODSIS [15] and based on modification indices, we correlated error terms between items 1 and 2 (see Figure 2.).

A significant Chi-Square difference test: *x*^2^(1) = 54.87; *p* < 0.001 and model fit indices supported the solution with the correlated residuals. The highest correlation between the residuals of manifest variables was *r* = 0.04.

### 3.3. Invariance Testing and Factor Loadings

The testing of the configural, metric and scalar invariance in both the OASIS and the ODSIS between genders revealed only marginal changes in the *CFI*, suggesting the equivalence of measurement of the OASIS and the ODSIS items (see Table 2). The factor loadings in the Overall model were high in the OASIS (from 0.75 to 0.88) and also in the ODSIS (from 0.86 to 0.92).

### 3.4. Internal Consistency and Test–Retest Reliability

The reliability of the OASIS was excellent: Cronbach’s α = 0.95, 95%CI (0.95–0.96) and McDonald’s ω_t_ = 0.95, 95%CI (0.95–0.95). Similarly to the previous scale, the ODSIS displayed an excellent internal consistency: Cronbach’s *α* = 0.95, 95%CI (0.95–0.96); McDonald’s *ωt* = 0.95, 95%CI (0.95–0.95). The intraclass correlations based on a two-way random effects model revealed that the OASIS: *r* = 0.83, 95%CI (0.17–0.96), *p* = 0.014 and the ODSIS: *r* = 0.85, 95%CI (0.29–0.97), *p* = 0.008 scores were relatively stable after one week.

### 3.5. Convergent Validity

Table 3 presents the bivariate zero-order correlations between the main study variables. The OASIS was positively correlated with depression, neuroticism and negative emotions experienced in the past week and was negatively correlated with self-esteem and age. Similarly, as in the previous scale, the ODSIS was positively correlated with neuroticism, anxiety and negative emotions experienced in the past week, and negatively correlated self-esteem and age. In the retest sample, a positive association was found between the OASIS and the GAD-7: *r_s_* = 0.87, *S* = 15.43, *p* = 0.002 and PHQ-9: *r_s_* = 0.79, *S* = 24.70, *p* = 0.011. When estimating the correlations of the ODSIS with the GAD-7 and the PHQ-9, Pearson correlations were used due to a low power: the ODSIS score positively correlated with the GAD-7: *r* = 0.81, 95%CI (0.32, 0.96), *t*(7) = 3.69, *p* = 0.008 and the PHQ-9: *r* = 0.84, 95%CI (0.39, 0.97), and *t*(7) = 4.08, *p* = 0.005.

The first ANCOVA indicated that females scored significantly higher on anxiety with a medium effect size: F(1, 1391) = 37.97, *MSE* = 17.59, *p* < 0.001, η^2^= 0.027, and also in depression with a small effect size: F(1, 1391) = 11.81, *MSE* = 18.73, *p* = 0.001, η^2^ = 0.008. After adding neuroticism as a covariate, the difference between males and females in anxiety, F(1, 1384) = 26.39, *MSE* = 12.41, *p* < 0.001, η^2^ = 0.019, and depression, F(1, 1384) = 4.01, *MSE* = 13.72, *p* = 0.045, η^2^ = 0.003, remained significant.

### 3.6. Sensitivity, Specifity and Cut-Off

**Figure 3:**
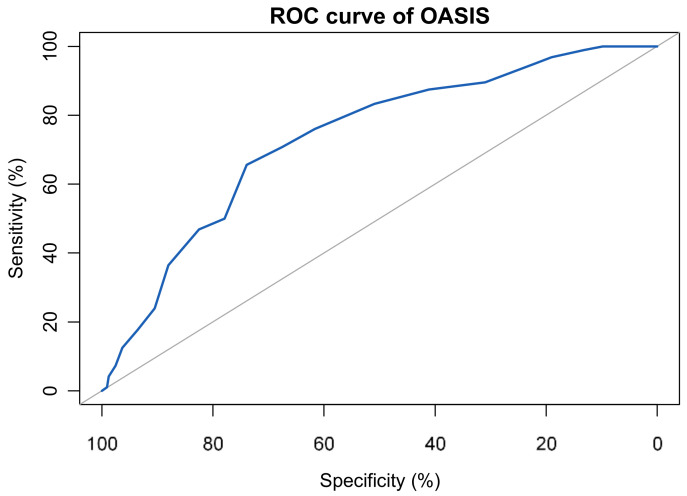
ROC curve of the OASIS (Sample 4, *n* = 494).

**Figure 4:**
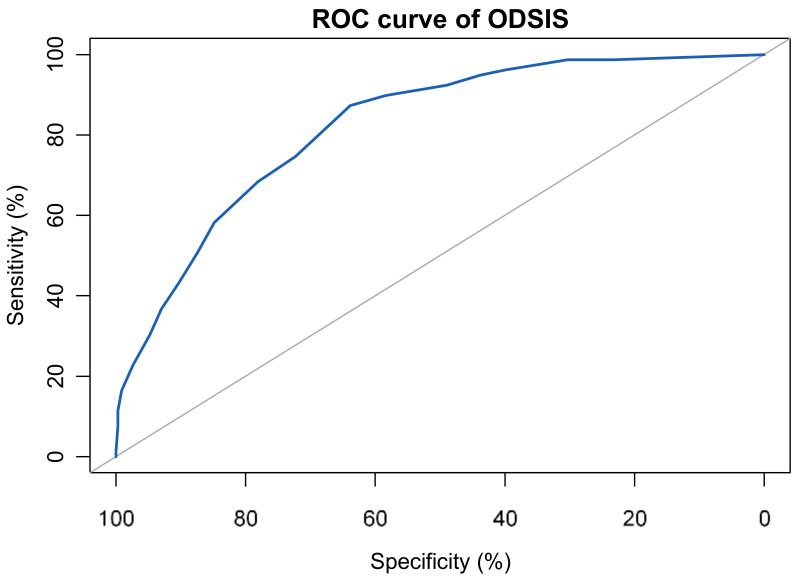
ROC curve of the ODSIS (Sample 4, *n* = 494).

The ROC suggested that the area under the curve for the OASIS was 0.73, 95%CI (0.68–0.79), and for the ODSIS was 0.82, 95%CI (0.78–0.87)—see Figure 3 and Figure 4. The result of the Youden index for the OASIS (0.4) and the ODSIS (0.51) suggested an optimal cut-off of 15 for the OASIS and 12 for the ODSIS. Using these cut-offs, the sensitivity and specificity were 0.66 and 0.74 for the OASIS and 0.87 and 0.64 for the ODSIS. When these cut-offs were used, 66% of individuals suffering from anxiety and 75% with depression were correctly identified. See Figure 3 and Figure 4 for ROC curves of the OASIS and the ODSIS.

## 4. Discussion

The purpose of this study was to psychometrically evaluate the Czech versions of the abbreviated OASIS and ODSIS. The study evaluated the validity and structure of both tools as well as their reliability and sensitivity. It also identified the cut-off scores. The psychometric properties of both scales were very good. The study identified differences in the levels of anxiety and depression among different groups of respondents.

In our study, we found a significantly higher level of anxiety and depression in students. As Wang et al. [3] stated, little is known about the impact of the current COVID-19 pandemic on the levels of depression and anxiety. However, a number of studies show elevated levels of anxiety and depression among students during the pandemic worldwide (e.g., [46,47,48,49,50]) and some studies indicate that the highest levels of mental discomfort appear in younger generations (e.g., [51]). These results suggest that these higher levels of anxiety and depression might be related to the ongoing COVID-19 pandemic and may be linked to uncertainty regarding the current and future situation. As employed and retired people in the Czech Republic have a steady and regular income, the uncertain financial situation of students would be a feasible explanation, but certainly not a complete one, of this difference. Other factors that influence the state of mental health of students are represented by social isolation and worries about one’s health, future career, family and friends, as identified in a study by Elmer et al. [50]. Some studies recommend the close monitoring of mental health in students during future pandemic situations to be able to provide preventive measures and professional help (e.g., [49]). In this context suicidal ideation also seems to be an important issue to address; neither OASIS nor ODSIS include items on this matter. As individuals with anxiety and depression might exhibit suicidal ideation, we recommend examining the possibility of adding one more item on suicidality and/or suicidal ideation to ODSIS in future research.

Next, we found that the respondents who identified themselves as religious showed a higher level of anxiety and depression when compared to the non-religious and atheist particpants. This was contrary to the findings of Dlugosz [51], who suggested that mental health improved with an increase in religious practices. Generally, studies regarding the relation between anxiety, depression and religiosity showed mixed and often contradictory results [52,53]. Some researchers, such as Ellis and Wahab [54] or Jong et al. [55], suggested that the association between death, anxiety and religiosity might be curvilinear rather than linear. According to the curvilinearity theory, people who are either strongly religious or strongly non-religious may be less anxious than those with ambivalent beliefs. Strongly religious individuals believe in an afterlife and are more likely to live according to the commandments of their religious system. Non-religious people reject the idea of a life-after-death. Consequently, both groups should have no reason to fear death and the afterlife. In contrast to the non-religious particpiants, moderately religious people might fear death more, as they are either not sure about the afterlife or do not live according to the standards of their religion. This might also explain the different findings regarding anxiety, depression and religiosity. However, further evidence is necessary to support this theory, as we are not able to distinguish the degree of religiosity based on the data collected for this study. The heterogeneity in the degree of religiousness may also be caused by measurement problems, as reported by Malinakova et al. [56]. Furthermore, as the previously mentioned study identified, the data may be distorted by the fact that in problematic times, a shift towards religiosity might occur in the secular environment by otherwise non-religious respondents.

The invariance testing of the OASIS and the ODSIS revealed that men and women responded similarly to both measures. Their answers did not differ significantly regarding the total score and individual items. The measurement invariance for men and women was reported only by Moore et al. [23] for the OASIS. However, female respondents scored significantly higher in both measurements. These findings are nevertheless consistent with the facts stated by the WHO [4]; that depression and anxiety disorders are more common among females than males.

Regarding the internal structure of the OASIS and the ODSIS, confirmatory factor analysis revealed a good fit for the unidimensional factor model with high factor loadings, as also shown in previous studies [12,13,14,15,16,17,19,21,22,23,24]. Similar to previous studies [15,22,23], we revealed a residual correlation between items one and two of both scales, and the one-factor model showed a satisfactory fit only after the error variance indices were allowed to correlate. However, the correlations between items one and two of both tools are logical because item one asks about the frequency of the symptoms during the past week and item two asks about their intensity. Consequently, if there were no symptoms, the intensity must also be zero. Due to the unidimensional structure, the use of the total score is duly justified as an indicator of the level of anxiety and depression.

The internal consistency and validity of both the OASIS and the ODSIS were very good. The correlations of both tools with instruments used to determine validity were significant and moved in the anticipated direction. The only exception was the low but positive correlation of both tools with the PANAS-P measuring positive affects. The reason for this may be that some items of the positive affects scale of the Czech version of the PANAS might not be perceived as being clearly positive (e.g., being “determined” or “attentive”). Another possible explanation was that, even though respondents experienced positive affects, the level of depression and anxiety remained high as a result of the present COVID-19 pandemic.

The cut-off value was initially determined to be ≥8 for both the OASIS [13,22,23] and the ODSIS [19] in the original English scales. The cut-off score of the different language versions of both tools varied between 5 and 11 [15,16,17,20,21]. In the Czech version, a cut-off value of 15 was identified for the OASIS (66% of correct classification) and a cut-off value of 12 for the ODSIS (75% of correct classification). Both values were relatively high compared to the values identified in the studies regarding the original versions or the other language variations of the tools. According to the results of Bragdon et al. [24], a value greater of than 12 in the OASIS indicated severe illness. As noted in previous studies, the differences in the cut-off score can be attributed to the differences in the sample characteristics [15,16]. The cut-off values of the Czech version might be influenced to a great extent by the ongoing pandemic situation and, consequently, are to be used and interpreted with caution.

### 4.1. Strengths and Limitations

The first strength of the study was that it was based on a large sample of Czech adults, which was well-balanced regarding age and gender of the respondents, so the characteristics were close to those of a nationally representative sample. Second, the study confirmed the strong psychometric properties of both tools. Third, the study represented the first validation of the OASIS and the ODSIS in the Czech language environment.

This study also had several limitations. First, as the study used a self-report approach for collecting data, the results might be influenced by the effects of socially desirable responding. Second, the data used in the study were cross-sectional. As a result, we could not make conclusions on causality regarding the different levels of anxiety and depression among the groups of respondents. Third, the examination of the relationship of the OASIS and the ODSIS with two of the measures for anxiety and depression was completed on a small sample of respondents. Therefore, we recommend re-examining these relationships on a larger sample in the future. Fourth, the data were collected during the COVID-19 pandemic, which could have influenced the identified cut-off scores. Fifth, despite the violated normality assumption, the Pearson correlation coefficient was used in the two analyses. This was completed due to the low statistical power in the two variables. As the normality assumption of our data was broken, the Pearson correlation could provide inaccurate results. For this reason, the associations of the ODSIS with the depression measures where the Pearson correlation coefficient was used should be interpreted with caution.

### 4.2. Implications

In our study, we found that the OASIS and the ODSIS were solid and effective tools for assessing the intensity of depression and anxiety, as well as the resulting impairment. The validation of the OASIS and the ODSIS in the Czech language may positively influence the early and easy identification of anxiety and depression in the general population and monitor the changes in intensity of symptoms. The findings show that it is necessary to address anxiety and depression as possible consequences of the current COVID-19 pandemic and their impact on mental and physical health of the general population.

OASIS and ODSIS can prove to be helpful in clinical settings as broad and time-effective screening instruments for symptoms and functional, as well as behavioral, impairments resulting from anxiety and mood disorders after the cut-off values are assessed in a clinical setting. The tools can save the time and energy of both the clients and the clinical professionals if the cut-off levels are not reached. The clients with levels of anxiety and depression over the cut-of points can undergo further diagnostic processes using more detailed and complex tools and measures to identify the nature of the problem and to set the right diagnosis.

The scales can be effectively used for research purposes to examine the associations between levels of depression and anxiety and the behavioral, emotional and cognitive changes in respondents. Further research regarding the differences in the levels of depression and anxiety between different groups in the Czech Republic is necessary. In order to be able to interpret the identified variations correctly, the influence of the positive and negative religious coping of individuals on depression and anxiety should be examined.

We recommend examining the cut-off values of the Czech versions of the tools once again in the future, after the COVID-19 pandemic ends. Similarly, the cut-off values for clinical use must be identified by future research.

## 5. Conclusions

The presented study succeeded in validating the Czech versions of the OASIS and the ODSIS. These language variations of the scales represent brief, 5-item, self-reporting tools for measuring anxiety, depression and the resulting impairment in the Czech population. The one-factor structure of both tools is confirmed, and the study also supports the internal consistency and construct validity of both tools. The OASIS and the ODSIS are suitable for screening and assessing anxiety and depression for research purposes.

## Figures and Tables

**Table 1:** Sociodemographic results of the samples.

Variable	Sample 1	Sample 2	Sample 3	Sample 4	OASIS Differences	ODSIS Differences
Gender						
1. Male	702 (50%)	503 (50%)	3 (30%)	85 (17%)		
2. Female	691 (50%)	512 (50%)	7 (70%)	409 (83%)		
Family status						
1. No relationship/widowed/divorced	476 (34%)	519 (51%)	2 (20%)	332 (67%)		
2. In a relationship/married	917 (66%)	496 (49%)	8 (80%)	162 (33%)		
Education						
1. Elementary school/vocational school	749 (54%)	469 (46%)		28 (5.7%)		
2. High school or higher vocational school (HVS)	467 (34%)	374 (37%)	4 (40%)	221 (45%)		
3. HSV or University bachelor		37 (3.7%)	5 (50%)	107 (22%)		
4. University master’s or higher		132 (13%)	1 (10%)	138 (28%)		
5. University unspecified	177 (13%)					
Economic status						
1. Working	754 (54%)	551 (54%)		265 (54%)		
2. Not working	124 (8.9%)	90 (8.9%)				
3. Pensioner	438 (31%)	318 (31%)			5 > 4 (*p* = 0.008, *Ȃ* = 0.35)	5 > 4 (*p* = 0.010, *Ȃ* = 0.35)
4. Student	77 (5.5%)	53 (5.2%)		143 (28%)	5 > 1 (*p* = 0.008, *Ȃ* = 0.36)	5 > 1 (*p* = 0.011, *Ȃ* = 0.36)
5. Other				86 (17%)		
Faith						
1. No, I am a convinced atheist	179 (13%)		1 (10%)	47 (9.6%)	1 < 4 (*p* = 0.014; *Ȃ* = 0.4), 1 < 3 (*p* = 0.031; *Ȃ* = 0.57),	
2. Non-religious	731 (52%)		4 (40%)	182 (37%)		
3. Yes, but I am not a member of church/religious society	362 (26%)		3 (30%)	199 (41%)	3 > 2 (*p* = 0.001, *Ȃ* = 0.43)	3 > 2 (*p* = 0.006, *Ȃ* = 0.42)
4. Yes, I am a member of church/religious society	121(8.7%)		2 (20%)	62 (13%)	5 > 6 (*p* = 0.002, *Ȃ* = 0.4)	

Note. *Ȃ* = represents effect size; sociodemographic differences are calculated based on data from the largest sample, i.e., Sample 1. The last two columns represent differences in the OASIS and the ODSIS between sociodemographic groups.

**Table 2:** OASIS and ODSIS measurement invariance models (Sample 1, *n* = 1393).

Model	*×* ^2^	df	*p*-Value	*CFI*	*TLI*	*RMSEA*	*RMSEA* 90%CI *lb*	*RMSEA* 90%CI *ub*	*SRMR*
OASIS									
Overall model	38.276	4	*p* < 0.001	0.999	0.997	0.078	0.057	0.102	0.027
Male model	26.276	4	*p* < 0.001	0.999	0.997	0.089	0.059	0.123	0.031
Female model	11.251	4	*p* = 0.024	0.999	0.999	0.051	0.017	0.088	0.022
Configural model	37.527	8	*p* < 0.001	0.999	0.998	0.073	0.05	0.097	0.027
Metric model	40.716	12	*p* < 0.001	0.999	0.999	0.059	0.039	0.079	0.028
Scalar model	74.638	26	*p* < 0.001	0.999	0.999	0.052	0.038	0.066	0.027
Strict model	74.638	26	*p* < 0.001	0.999	0.999	0.052	0.038	0.066	0.027
ODSIS									
Overall model	36.538	4	*p* < 0.001	0.999	0.999	0.076	0.055	0.1	0.021
Male model	28.207	4	*p* < 0.001	0.999	0.998	0.093	0.062	0.127	0.026
Female model	10.107	4	*p* = 0.039	1	1	0.047	0.01	0.084	0.016
Configural model	38.314	8	*p* < 0.001	0.999	0.999	0.074	0.051	0.098	0.021
Metric model	49.469	12	*p* < 0.001	0.999	0.999	0.067	0.048	0.087	0.023
Scalar model	52.548	26	*p* = 0.002	1	1	0.038	0.023	0.053	0.022
Strict model	52.548	26	*p* = 0.002	1	1	0.038	0.023	0.053	0.022

Note. ×2 = Chi-Square, df = degrees of freedom, *CFI* = Comparative Fit Index, *TLI* = Tucker–Lewis Index, *RMSEA* = Root Mean Square Error of Approximation, *CI* = Confidence Interval, *lb* = lower bound of the 90% confidence interval, *ub* = upper bound of the 90% confidence interval, *SRMR* = Standardized Root Mean Square Residual.

**Table 3:** Correlation table with means and standard deviations (Sample 4, *n* = 494).

	1	2	3	4	5	6	7	8	9	*M*	*SD*
1. OASIS	-									9.50	4.25
2. ODSIS	0.83 ***	-								8.73	4.34
3. Gender	0.17 ***	0.09 ***	-							1.50	0.50
4. BFI_N	0.54 ***	0.51 ***	0.09 **	-						2.76	0.73
5. RSES	−0.40 ***	−0.42 ***	−0.05	−0.53 ***	-					28.89	4.69
6. PANAS-P	0.23 ***	0.20 ***	−0.02	0.07 **	0.03	-				28.83	9.77
7. PANAS-N	0.56 ***	0.53 ***	0.06 *	0.45 ***	−0.30 ***	0.61 ***	-			18.27	7.58
8. Age	−0.11 ***	−0.16 ***	0.08 **	−0.22 ***	0.21 ***	−0.16 ***	−0.17 ***	-		48.29	16.42
9. Education	0.01	−0.03	0.20 ***	−0.01	0.07 *	0.08 **	0.06 *	−0.07 **	-	2.51	0.82
10. Faith	0.09 ***	0.08 **	0.05	0.02	−0.01	0.04	0.01	0.04	0.09 **	1.78	0.98

Note. * *p* < 0.05; ** *p* < 0.01; *** *p* < 0.001; *M* = mean; *SD* = standard deviation; OASIS = Overall Anxiety and Impairment Scale; ODSIS = Overall Depression and Impairment Scale; BFI_N = Big Five Inventory–Neuroticism subscale; RSES = Rosenberg Self-Esteem Scale; *PANAS-P* = The Positive and Negative Affect Schedule–Positive emotions subscale; *PANAS-N* = The Positive and Negative Affect Schedule–Negative emotions subscale. Correlations were calculated using Spearman rank correlation coefficient.

## Data Availability

Data are available at: https://osf.io/ad6b3/.
